# Nano-particle coated or impregnated acrylic resins in dental applications: A systematic review of in Vivo Evidence on mechanical properties, biocompatibility and clinical performance

**DOI:** 10.1016/j.jobcr.2025.07.018

**Published:** 2025-08-07

**Authors:** Parthasarthy Natarajan, Seenivasan Madhan Kumar, Shanmuganathan Natarajan, Dr K.S. Sridharan, Dr S. Narayana Kalkura

**Affiliations:** a Sri Ramachandra Dental College and Hospital, India; b Department of Microbiology, Sri Ramachandra Institute of Higher Education and Research, India; c Crystal Growth Centre, Anna University, India

**Keywords:** Nanoparticles, Acrylic resin, Polymethyl methacrylate, Antimicrobial activity, Biocompatibility, Mechanical strength, Esthetic properties, Denture base materials, Systematic review

## Abstract

**Background:**

Acrylic resins are extensively used in prosthodontics, orthodontics and maxillofacial prosthetics due to their ease of fabrication and cost-effectiveness. However, conventional acrylic materials are susceptible to microbial colonization, mechanical deterioration and esthetic compromise. To overcome these limitations, recent research has explored the incorporation of nanoparticles into polymethyl methacrylate (PMMA)-based resins to enhance their antimicrobial efficacy, mechanical strength, biocompatibility, and long-term durability.

**Methods:**

This systematic review was conducted according to PRISMA guidelines. An extensive literature search was performed across PubMed/MEDLINE, Scopus, Web of Science, Cochrane Library and Embase for studies published up to January 18, 2025. Only in vivo studies conducted on humans or animals evaluating nanoparticle-coated or nanoparticle-impregnated acrylic resins were included. Standalone in vitro studies were excluded. Risk of bias was assessed using Cochrane's RoB 2.0 tool for randomized controlled trials (RCTs), ROBINS-I for non-randomized studies and the SYRCLE tool for animal studies.

**Results:**

Out of 3154 records initially identified, six studies met the eligibility criteria. The nanoparticles incorporated included silver, titanium dioxide, nanocopper, nanogold and quaternary ammonium polyethyleneimine (QPEI). All included studies reported antimicrobial activity with nanogold, nanocopper and QPEI showing sustained microbial inhibition. Mechanical outcomes varied: silver and titanium dioxide nanoparticles were associated with reduced material strength, whereas nanocopper maintained mechanical performance. Esthetic outcomes indicated that silver-based modifications caused discoloration, while nanocopper and QPEI preserved color stability.

**Conclusion:**

Nanoparticle-modified acrylic resins exhibit enhanced antimicrobial and biocompatibility profiles with certain formulations particularly those incorporating nanocopper, nanogold and QPEI showing greater clinical potential. However, mechanical durability and esthetic alterations remain challenges especially with silver and titanium-based additives. Further well-designed, long-term randomized controlled trials are warranted to validate the clinical applicability of these nano-enhanced acrylic materials.

## Graphical abstract

**Figure:**
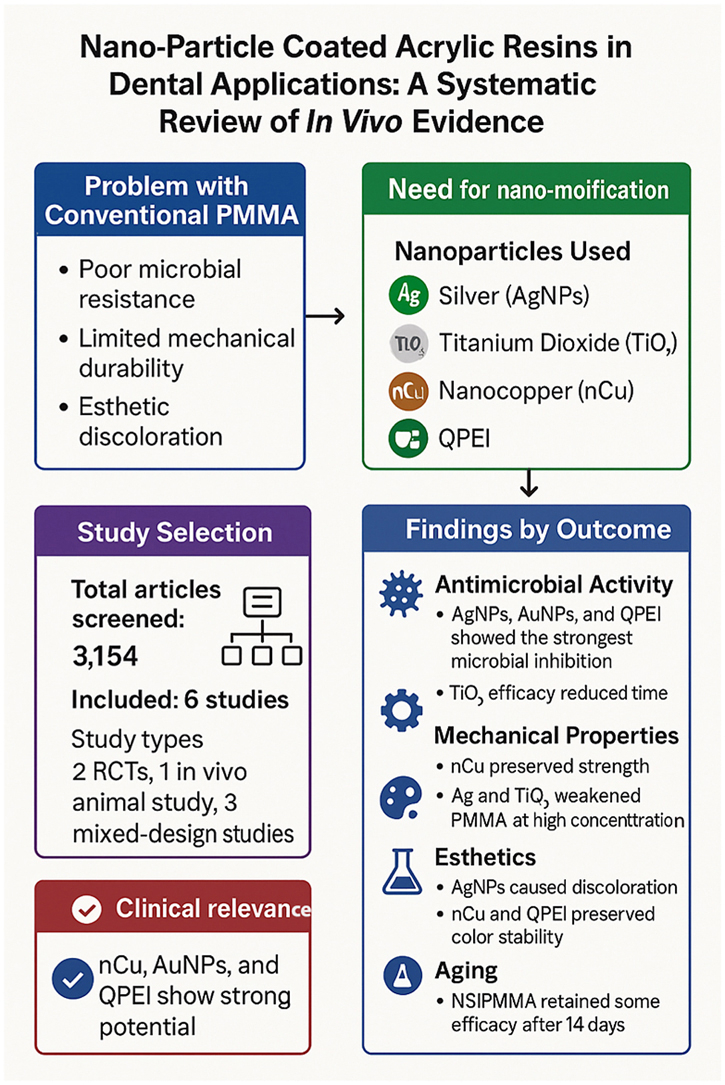

## 1 Introduction

Acrylic resins which are widely used in prosthodontics, orthodontics and maxillofacial prosthetics offer advantages such as ease of fabrication, cost-effectiveness and esthetic appeal.^1^ However, they also have limitations which include low fracture resistance, microbial adhesion, esthetic degradation and potential cytotoxicity caused by the release of residual monomers.^2^^,^^3^ To overcome these drawbacks, researchers have focused on modifying acrylic resins by incorporating nanoparticles. This approach enhances mechanical strength, antimicrobial properties and biocompatibility while maintaining esthetic stability.^4^ Nanotechnology, which plays a significant role in biomaterials research, has introduced nano-fillers, coatings and impregnations to improve the functional properties of acrylic resins.^5^ Different nanoparticles such as silver (Ag), titanium dioxide (TiO_2_), zirconia (ZrO_2_), silica (SiO_2_) and graphene oxide (GO) have been studied for their ability to enhance the structural and biological characteristics of these materials.^6^

The mechanical properties of acrylic resins which include fracture toughness and flexural strength determine their durability in clinical applications.^7^ By reinforcing these resins with nanoparticles such as zirconia and silica, researchers have significantly improved their resistance to mechanical stress.^8^ This enhancement which is achieved by increasing polymer cross-linking and reducing porosity extends the longevity of prosthetic appliances.^9^ Another major concern of acrylic resins is their susceptibility to microbial colonization. The adhesion of microorganisms, such as *Candida albicans* and *Streptococcus mutans* to acrylic surfaces can lead to complications like denture stomatitis and secondary caries.^10^ To address this issue, researchers have incorporated antimicrobial nanoparticles, such as silver, titanium dioxide and zinc oxide. These nanoparticles inhibit microbial adhesion by disrupting biofilm formation, which helps in reducing infection risks associated with prosthetic devices.^11^ However, the use of certain nanoparticles raises concerns regarding cytotoxic effects.^3^ Some nanoparticles may induce oxidative stress and inflammatory responses, which can negatively affect surrounding tissues. While in vitro studies have demonstrated promising antimicrobial effects, their relevance in clinical settings remains uncertain.^12^, ^13^, ^14^ This highlights the need for more in vivo investigations, which provide a more accurate assessment of the biological interactions of these materials.

Esthetic properties which play a crucial role in patient satisfaction can be affected by prolonged exposure to oral fluids, staining agents and mechanical wear.^15^^,^^16^ This leads to discoloration and surface degradation, which compromise the overall appearance of prosthetic restorations.^17^ By incorporating nanoparticles such as titanium dioxide and silica, researchers have improved the colour stability, gloss retention, and surface smoothness of acrylic resins.^18^ These modifications, which enhance long-term esthetic outcomes, make prosthetic appliances more durable and visually appealing. Despite these advancements, translating laboratory findings into clinical applications remains a challenge. In vivo studies, which are essential for assessing biological interactions, provide insights into tissue compatibility, degradation behaviour, and potential immunogenic responses.^19^, ^20^, ^21^ While several in vitro studies have explored the effects of nanoparticles on acrylic resins, their findings may not fully reflect real-world performance.^12^^,^^14^^,^^22^ Clinical studies which focus on human and animal models, are necessary to validate these laboratory results.

Given the increasing use of nano-engineered acrylic resins in dentistry, it is important to systematically evaluate available in vivo evidence. Previous reviews have primarily focused on in vitro studies, which provide useful preliminary data but may not accurately represent clinical conditions.^23^, ^24^, ^25^ To bridge this gap, the present systematic review aims to assess human and animal studies on nano-particle coated or impregnated acrylic resins. By examining mechanical properties, biocompatibility, antimicrobial activity, esthetic outcomes, material aging and potential allergic or inflammatory responses, this review seeks to provide clinically relevant insights. Standalone in vitro studies have been excluded, which ensures a focus on real-world clinical applications.

## 2 Materials and methods

This systematic review was conducted in accordance with the PRISMA (Preferred Reporting Items for Systematic Reviews and Meta-Analyses) guidelines, ensuring transparency, reproducibility, and methodological rigor.^26^

### 2.1 Registration and protocol

The review protocol was not prospectively registered in any database. No pre-specified protocol was publicly accessible at the time of conducting this review.

### 2.2 PICO question

The review question was framed using the PICO framework: "In human and animal models (P), does the use of nano-particle coated or impregnated acrylic resins (I), compared to conventional acrylic resins without nano-modification (C), improve mechanical properties, biocompatibility, antimicrobial activity, esthetics and resistance to material aging and reduce allergic or inflammatory responses (O)?"

### 2.3 Search strategy

A comprehensive literature search was performed across five electronic databases: PubMed/MEDLINE, Scopus, Web of Science, Cochrane Library and Embase for studies published from database inception to January 18, 2025. To maximize sensitivity and specificity, a combination of Medical Subject Headings (MeSH) terms, free-text keywords, and Boolean operators (AND, OR, NOT) was applied. Search terms included "acrylic resin," "polymethyl methacrylate (PMMA)," "denture base resin," "nano-particle," "nano-coating," "nano-impregnation," "mechanical properties," "biocompatibility," "antimicrobial activity," "material aging," and "allergic reactions," among others (Supplementary Table 1) Additionally, grey literature sources such as ProQuest Dissertations & Theses were screened. A manual search of the reference lists of selected articles and relevant systematic reviews was conducted to ensure comprehensive literature coverage.

### 2.4 Study selection

Study selection was carried out based on predefined eligibility criteria structured around the PICO framework. The population included human and animal models evaluating nano-particle coated or impregnated acrylic resins. The intervention involved the use of nano-particles in acrylic resins, either as coatings or as incorporated materials, while the comparator group consisted of conventional acrylic resins without nano-modification. The primary outcomes of interest were mechanical properties, biocompatibility, antimicrobial activity, esthetic outcomes, material aging, and allergic or inflammatory responses. Inclusion criteria required that studies assess at least one of these predefined outcomes in vivo, providing either quantitative or qualitative data for comparative analysis.

Studies were eligible if they: (1) included human or animal models, (2) evaluated acrylic resins coated or impregnated with nanoparticles, (3) compared them with unmodified acrylic resins, and (4) reported outcomes on mechanical properties, biocompatibility, antimicrobial activity, esthetics, resistance to material aging, or allergic/inflammatory responses. Only in vivo studies or those with both in vivo and in vitro components were included. Eligible study designs comprised randomized controlled trials (RCTs), controlled clinical trials (CCTs), cohort and case-control studies. Articles had to be in English.

Studies were excluded if they were standalone in vitro studies, reported on non-dental applications of modified PMMA were review articles, editorials, commentaries or conference abstracts. Conference abstracts were excluded due to lack of detailed methodology and peer review which compromises reproducibility and comprehensive appraisal. Moreover, conference abstracts often do not undergo updates post-publication, which may limit access to final results.

The study selection process was performed in two stages. In the first stage, two independent reviewers screened the titles and abstracts of all identified records. Articles that clearly did not meet the eligibility criteria were excluded at this stage. In the second stage, the full texts of potentially relevant studies were retrieved and reviewed in detail for final inclusion. Disagreements between reviewers were resolved through discussion, and in cases where consensus could not be reached, a third reviewer was consulted. To ensure a transparent selection process, a PRISMA flow diagram was used to document the number of records identified, screened, excluded, and finally included in the systematic review.

### 2.5 Data extraction

The data extraction process was carried out systematically to ensure accuracy and consistency in the collection of relevant study information. A standardized data extraction form was developed to capture key details from each included study. The extracted data fields included the first author and year of publication, study design, sample size, sample characteristics, compared groups, mechanical properties, cytotoxicity, antimicrobial activity, esthetics, material aging test, observations of the animal study and inference.

Two independent reviewers performed data extraction, with discrepancies resolved through discussion or consultation with a third reviewer. The extracted data were first entered into a structured spreadsheet, which facilitated cross-verification of study characteristics and outcome measures. For each study, the first author and year were recorded to ensure proper citation and reference tracking. The study design was categorized as randomized controlled trials (RCTs), pilot trials, non-randomized controlled trials (non-RCTs), or animal studies, allowing for differentiation in methodological rigor. The sample size and sample characteristics were carefully documented, noting population demographics in human studies and species, strain, and experimental conditions in animal studies.

The compared groups were identified, detailing intervention and control groups, including variations in nano-particle coatings, acrylic resin formulations, or other modifications. Study outcomes related to mechanical properties were extracted, focusing on parameters such as flexural strength, fracture resistance, wear resistance, and surface roughness. The cytotoxicity data included assessments of biocompatibility, cell viability, and tissue response, while antimicrobial activity was recorded based on bacterial adhesion, biofilm formation, and microbial inhibition studies. The esthetic outcomes were extracted, including color stability, gloss retention, and surface integrity. Data on material aging tests were collected to assess long-term performance, degradation, and stability of nano-modified acrylic resins.

For the animal study, specific observations were recorded regarding experimental conditions, host tissue response, inflammatory reactions, and histological findings. Finally, the inference section summarized the overall findings of each study, emphasizing the impact of nano-particle coatings or impregnations on acrylic resins.

The extracted data were reviewed to ensure completeness and consistency. If any missing or unclear data were identified, attempts were made to contact the study authors for clarification. The final dataset was structured in tabular form for systematic analysis, facilitating a transparent synthesis of study characteristics and outcomes.

### 2.6 Quality assessment

The risk of bias assessment was conducted to evaluate the methodological quality of the included studies. For the four RCTs and the pilot trial, the Cochrane Risk of Bias (RoB 2) tool was used.^27^ This tool assesses the risk of bias across five domains: the randomization process, deviations from intended interventions, missing outcome data, measurement of the outcome, and selection of the reported result.

For the non-randomized controlled trial (non-RCT), the ROBINS-I (Risk Of Bias In Non-randomized Studies of Interventions) tool was employed.^28^ This tool assesses bias across seven domains: confounding, selection of participants into the study, classification of interventions, deviations from intended interventions, missing data, measurement of outcomes, and selection of the reported result.

For the animal study, the SYRCLE's Risk of Bias (SYstematic Review Center for Laboratory Animal Experimentation) tool was applied.^29^ This tool, adapted from the Cochrane RoB tool, evaluates bias in domains such as sequence generation, baseline characteristics, allocation concealment, blinding, incomplete outcome data, and selective reporting. Since animal studies may have differences in methodology compared to human trials, additional considerations were given to the extent of blinding, allocation procedures, and the handling of experimental conditions that could introduce bias.

Additionally, two human trials and one animal study included an in vitro component. The QUIN (Quality in In Vivo Evidence) tool was used to assess potential bias in the in vitro methodologies incorporated within these studies.^30^ The QUIN tool evaluates study design, sample randomization, blinding of assessors, outcome measurement techniques, and reproducibility of results.

All risk of bias assessments were performed independently by two reviewers, with disagreements resolved through discussion or consultation with a third reviewer. The results of the risk of bias assessment were tabulated and visually represented using a traffic-light plot to summarize the risk of bias across the included studies.

## 3 Results

### 3.1 Study selection

**Fig. 1:**
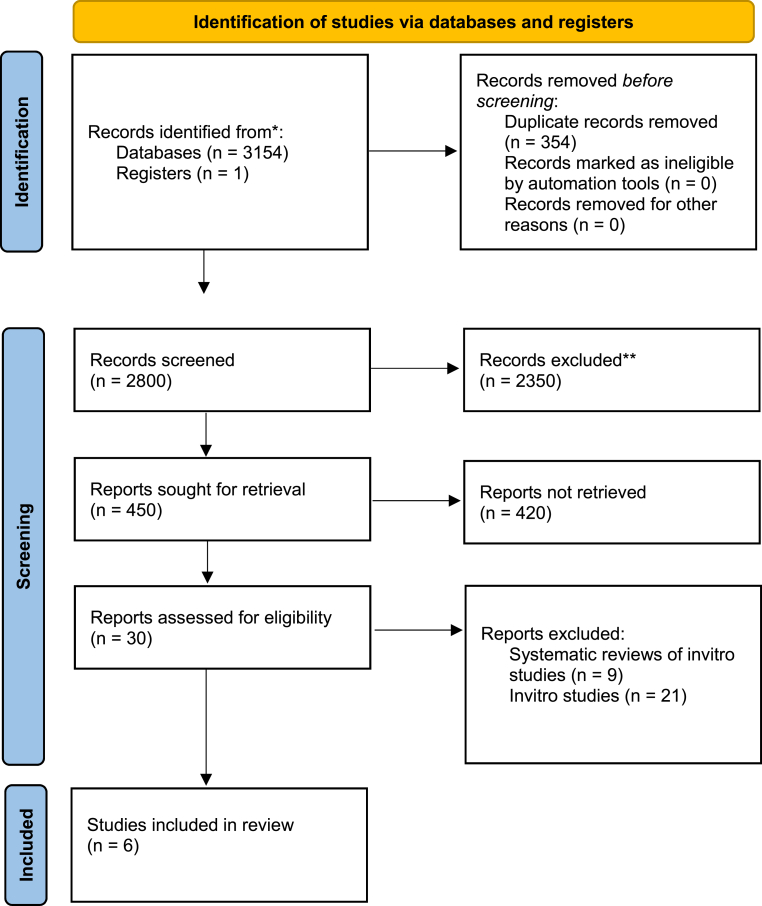
PRISMA flow diagram ∗Consider, if feasible to do so, reporting the number of records identified from each database or register searched (rather than the total number across all databases/registers). ∗∗If automation tools were used, indicate how many records were excluded by a human and how many were excluded by automation tools. Source: Page MJ et al. BMJ 2021; 372:n71. doi: 10.1136/bmj.n71. This work is licensed under CC BY 4.0. To view a copy of this license, visit https://creativecommons.org/licenses/by/4.0/.

A total of 3154 records were retrieved through systematic database searches across PubMed/MEDLINE, Scopus, Web of Science, Cochrane Library, Embase, and Google Scholar. After removing 354 duplicates, 2800 articles were screened by title and abstract. Of these, 450 full texts were reviewed in detail. Ultimately, six studies met the eligibility criteria for inclusion in the qualitative synthesis (Fig. 1, Supplementary Table 2). One additional trial was identified in the WHO-ICTRP registry but was excluded due to its ongoing status and unavailability of results.^31^

### 3.2 Characteristics of included studies

Among the six included studies, two were randomized controlled trials,^34^^,^^35^ one was a randomized controlled study,^20^ one combined in vitro and in vivo animal research,^19^ one involved a preliminary clinical trial following in vitro analysis,^33^ and one was an RCT with in vitro components.^32^ Sample sizes ranged from 10 to 50 participants in human trials and 23 to 54 samples in experimental arms (Table 1, Table 2).

**Table 1:** Characteristics of included studies.

First author & year	Study design	Sample size	Sample characteristics	Compared groups	Mechanical properties
Jie Sun et al.2021^19^	In-vitro and In-vivo animal study	Invitro – 54, Mechanical properties −12, Animal study – 23	54 - disc shaped specimens, 12 rectangular samples, 20 BALB/c mice +3 New Zealand Big Eared white rabbit	(1):PMMA group (NAg-free); (2) NP/PMMA group (NAg powders (NP) mixed with PMMA powder then with MMA liquid); (3) NS/PMMA group (NAg solution was mixed with the acrylic acid and then with MMA liquid and PMMA powder).	Bending strength of NP/PMMA and NS/PMMA decreased than the ISO specifications (20795-1). Impact strength and elastic modulus were similar to control group
Eugenia Eftimie Totu et al., 2017^32^	Non RCT and Experimental in-vitro study	Not explicitly mentioned for each test, but multiple PMMA-TiO2 nanocomposite samples were tested	PMMA-TiO2 nanocomposite with TiO2 concentrations of 0.2 %, 0.4 %, 0.6 %, 1 %, and 2.5 % by weight	PMMA-TiO2 nanocomposites vs. pure PMMA (control)	Increasing TiO2 resulted in particle aggregation, altering viscosity and potentially lowering mechanical properties
Sebastian Correa et al., 2024^33^	Experimental in-vitro study and a preliminary clinical trial	50 participants in clinical trial; multiple nanocomposite samples tested in vitro	(25 nCu/PMMA, 25 PMMA)	nCu/PMMA vs. conventional PMMA denture wearers	No significant difference in flexural strength and modulus between nCu/PMMA and PMMA
Yasmin S. Zidan et al., 2025^34^	Randomized clinical trial	22 patients (11 in each group)	Completely edentulous male patients rehabilitated with mandibular overdentures retained by two dental implants	Conventional PMMA denture base (control) vs. PMMA denture base modified with nanogold particles	Not explicitly mentioned
Ghada M. Elabd et al., 2024^35^	Randomized controlled clinical trial	26 patients (13 in each group)	Orthodontic patients using twin block functional appliances with or without TiO2 nanoparticles in acrylic baseplates	TiO2-incorporated acrylic baseplate vs. conventional acrylic baseplate	Not explicitly mentioned
Nurit Beyth et al., 2010^20^	Randomized controlled study	10 volunteers	Healthy adult volunteers (aged 25–55 years) wearing a removable acrylic appliance with composite resin specimens	Resin composite with 1 % quaternary ammonium polyethyleneimine (QPEI) nanoparticles vs. conventional resin composite	Not explicitly mentioned

**Table 2:** Outcomes and clinical performance of assessed nanoparticle coated or impregnated acrylic resins.

First author & year	Cytotoxicity	Anti-microbial activity	Esthetics	Material aging test	Observations of the animal study	Inference
Jie Sun et al.2021^19^	Extracts from each group had no cytotoxicity towards L929 cells.	NP/PMMA and NS/PMMA denture bases against E.coli and Candida albicans were similar but antimicrobial effect against S.mutans was greatest.	Denture base specimens of NS/PMMA were orange yellow, NP/PMMA were tan.	After 14 days of accelerated aging, the antimicrobial effect of NP/PMMA group essentially disappeared. The antimicrobial effect of NS/PMMA towards the three stains also decreased but there was still clear inhibition zones	No sensitizations such as erythema, edema, ulcer or indurations were observed after injection, and after 24, 48 and 72 h	NS/PMMA denture base exhibits superior antimicrobial efficacy, aesthetics, biocompatibility, and mechanical properties, making it a promising material for clinical application.
Eugenia Eftimie Totu et al., 2017^32^	Not directly assessed, but biocompatibility tests are suggested for future research	0.4 %, 1 %, and 2.5 % TiO2 nanocomposites inhibited Candida scotti growth, with 0.4 % showing optimal effects	No specific assessment, but the final 3D printed denture was functionally and aesthetically suitable	FTIR analysis showed changes in the polymer matrix with TiO2 addition, suggesting modifications in long-term stability	No animal study performed	PMMA-TiO2 (0.4 %) nanocomposite showed antimicrobial properties, compatibility with stereolithographic 3D printing, and potential for denture manufacturing, but mechanical and biocompatibility tests are needed for clinical applications.
Sebastian Correa et al., 2024^33^	nCu/PMMA exhibited no cytotoxic effects on human gingival stem cells	nCu/PMMA showed strong antifungal effects against Candida albicans and inhibited biofilm formation	nCu/PMMA dentures retained esthetic properties similar to conventional PMMA	Copper ion release was minimal and within safe limits over 35 days in artificial saliva	No animal study performed	nCu/PMMA dentures demonstrated antimicrobial properties, biocompatibility, and reduced incidence of denture stomatitis, making them a promising alternative for preventing oral infections.
Yasmin S. Zidan et al., 2025^34^	Not directly assessed, but nanogold was chosen for its biocompatibility	PMMA-AuNPs showed significant inhibition of Candida albicans, Escherichia coli, and Streptococcus mutans at 2, 4, and 6 months	Not specifically discussed	Not explicitly mentioned, but microbial growth was assessed over 6 months	No animal study performed	Incorporation of gold nanoparticles into PMMA denture bases significantly reduced microbial adhesion and colonization compared to conventional acrylic resin bases in implant-retained overdentures
Ghada M. Elabd et al., 2024^35^	No allergic reactions, taste complaints, or visible adverse effects observed	Significant reduction in bacterial colony count under the baseplate after 4 and 6 months in the TiO2 group	Not specifically discussed	Not explicitly mentioned	No animal study performed	The addition of 1 % titanium dioxide nanoparticles to acrylic baseplates of orthodontic functional appliances significantly reduced bacterial colony counts, suggesting enhanced antimicrobial properties
Nurit Beyth et al., 2010^20^	No signs of inflammation, allergic reactions, or discomfort reported in volunteers	QPEI-modified resin composite showed >50 % reduction in bacterial vitality and significant inhibition of salivary biofilm growth	Not specifically discussed	No leaching of antibacterial nanoparticles, indicating stable long-term antibacterial properties	No animal study performed	Incorporation of QPEI nanoparticles in resin composite demonstrated significant in vivo antibacterial and antibiofilm activity, potentially improving longevity of dental restorations

Nanoparticles assessed included silver (AgNP),^19^ titanium dioxide (TiO_2_),^32^^,^^35^ nanocopper (nCu),^33^ nanogold (AuNPs)^34^ and quaternary ammonium polyethyleneimine (QPEI).^20^ All studies compared nano-modified PMMA to unmodified PMMA in terms of at least one of the following: mechanical performance, antimicrobial activity, biocompatibility, esthetics or material aging.

Four studies reported on mechanical properties^19^^,^^32^, ^33^, ^34^; all six evaluated antimicrobial efficacy.^19^^,^^20^^,^^32^, ^33^, ^34^, ^35^ Only two studies explicitly analyzed esthetic changes^19^^,^^33^ and only one study^19^ included in vivo animal assessment.

### 3.3 Risk of bias assessment

**Fig. 2:**
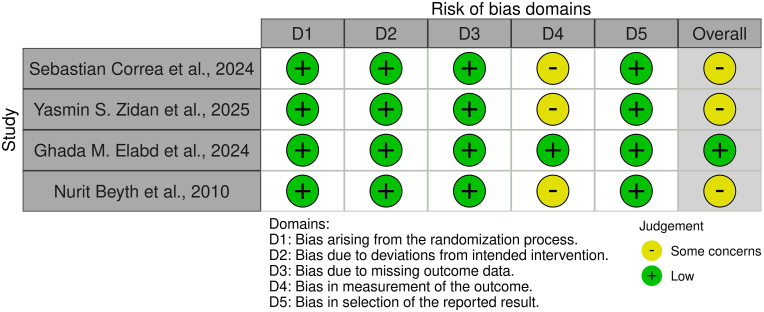
Risk of bias assessment using ROB 2.0 tool.

Risk of bias across studies was variable but generally moderate to low. Using ROB 2.0, four clinical trials^20^^,^^33^, ^34^, ^35^ were appraised. Most exhibited low risk in randomization, intervention adherence, and outcome reporting domains. However, three studies showed potential measurement bias due to unblinded assessments or subjective endpoints.^20^^,^^33^^,^^34^ Ghada M. Elabd et al., 2024^35^ was the only study with low risk in all ROB 2.0 domains (Fig. 2, Supplementary Fig. 1).

**Fig. 3:**
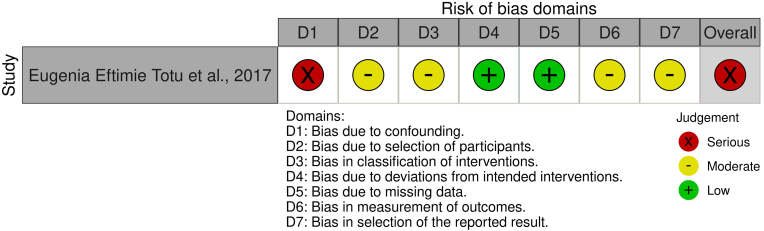
Risk of bias assessment using ROBINS-I tool.

The ROBINS-I assessment for Totu et al., 2017^32^ revealed moderate to serious risk, particularly due to unaddressed confounding and lack of allocation blinding. Outcome measurement and selective reporting were also rated at moderate risk (Fig. 3, Supplementary Fig. 2).

**Fig. 4:**
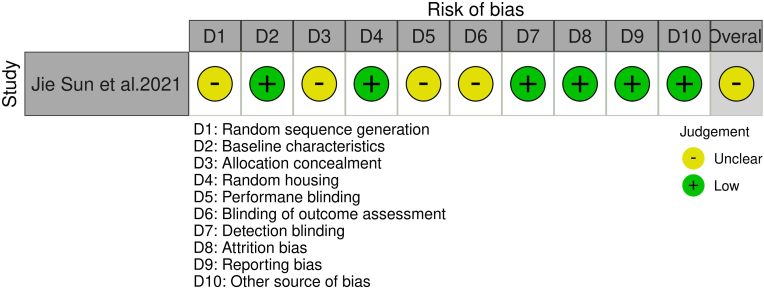
Risk of bias assessment using SYRCLE tool.

SYRCLE tool revealed moderate overall bias in the animal study by Sun et al. 2021,^19^ largely due to unclear randomization and blinding. Nonetheless, complete data reporting and standardized conditions were strengths (Fig. 4, Supplementary Fig. 3).

**Fig. 5:**
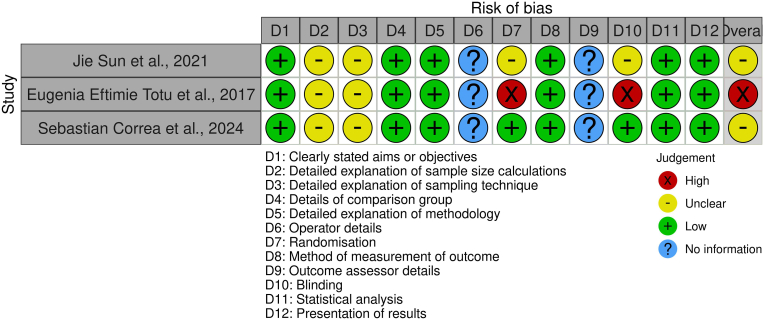
Risk of bias assessment using QUIN tool.

The in vitro components of three studies^19^^,^^32^^,^^33^ showed low risk in experimental clarity but had limitations in randomization and blinding. Totu et al., 2017^32^ scored highest in bias risk due to lack of randomization and methodological concealment (Fig. 5, Supplementary Fig. 4).

### 3.4 Narrative synthesis of findings

#### 3.4.1 Mechanical performance

Four studies assessed mechanical strength. Sun et al. 2021^19^ reported reduced bending strength in both NP/PMMA and NS/PMMA groups compared to ISO 20795-1 standards, though other mechanical indices remained unaffected. Totu et al., 2017^32^ observed deterioration in mechanical performance at higher TiO_2_ concentrations due to particle aggregation. In contrast, Correa et al., 2024^33^ found nCu/PMMA retained flexural strength similar to conventional PMMA supporting its mechanical viability (Table 2).

#### 3.4.2 Cytotoxicity and biocompatibility

Direct cytotoxicity assessments in three studies^19^^,^^20^^,^^32^ confirmed absence of adverse effects in cell cultures and human volunteers. Other studies inferred biocompatibility based on known nanoparticle profiles. No systemic or local toxicity was reported (Table 2).

#### 3.4.3 Antimicrobial efficacy

All six studies demonstrated enhanced microbial inhibition with nanoparticle-modified PMMA.^19^^,^^20^^,^^32^, ^33^, ^34^, ^35^ NS/PMMA showed the strongest effects against *S. mutans* and *C. albicans*.^19^ TiO_2_ formulations at ≥0.4 % were effective against *C. scotti*,^14^ while QPEI demonstrated a 50 % reduction in salivary biofilm viability.^20^ Clinical reductions in microbial load were also noted in Elabd et al., 2024^35^ and Zidan et al., 2025^34^ (Table 2).

#### 3.4.4 Esthetic properties

Only two studies evaluated esthetics.^19^^,^^33^ Sun et al. 2021^19^ reported yellow and tan discoloration in AgNP-containing PMMA. In contrast, Correa et al., 2024^33^ found no compromise in esthetic parameters, suggesting better optical compatibility with nCu (Table 2).

#### 3.4.5 Material aging and stability

Three studies reported on aging. Sun et al. 2021^19^ found antimicrobial activity in NP/PMMA diminished entirely after 14 days, while NS/PMMA retained partial effects. FTIR data from Totu et al., 2017^32^ indicated polymer matrix changes with TiO_2_. Copper ion release in Correa et al., 2024^33^ remained within safe limits over 35 days supporting its chemical stability (Table 2).

#### 3.4.6 In vivo observations

Only one animal study, Sun et al., 2021^19^ assessed host response to modified PMMA reporting no visible inflammation, erythema, or ulceration. The remaining studies were limited to human or in vitro assessments (Table 2).

#### 3.4.7 Quantitative synthesis

Meta-analysis was not performed due to high heterogeneity in nanoparticle types, outcome metrics, PMMA modifications and experimental designs. The variation in study methodologies and endpoints would have rendered statistical pooling inappropriate. Instead, a narrative synthesis was conducted following SWiM guidelines to ensure transparent, structured reporting.

## 4 Discussion

The findings of this systematic review suggest that nano-particle coated or impregnated acrylic resins offer significant advantages in terms of antimicrobial activity, biocompatibility, and material performance. However, variations in mechanical properties, esthetic outcomes, and long-term stability indicate that further optimization and standardization are required before these materials can be widely adopted in clinical practice. The reviewed studies investigated different nanoparticles, including silver (NAg), titanium dioxide (TiO_2_), nanocopper (nCu), nanogold (AuNPs), and quaternary ammonium polyethyleneimine (QPEI), each exhibiting unique characteristics that influence the overall clinical applicability of nano-modified acrylic resins.

### 4.1 Antimicrobial properties and clinical implications

One of the most promising findings of this review is the significant antimicrobial activity exhibited by nano-modified PMMA resins. All six included studies reported some degree of microbial inhibition, with nanoparticles demonstrating efficacy against Candida albicans, Streptococcus mutans, Escherichia coli, and biofilm formation.^19^^,^^20^^,^^32^, ^33^, ^34^, ^35^

Among these, silver nanoparticles (NAg) and nanogold (AuNPs) exhibited the strongest antimicrobial activity, with Jie Sun et al. (2021) showing that NS/PMMA had the greatest inhibition of *S. mutans*, while Yasmin S. Zidan et al. (2025) reported that PMMA-AuNPs reduced microbial adhesion for up to six months.^19^^,^^34^ Nanocopper (nCu) and QPEI nanoparticles also showed strong antimicrobial properties, with Sebastian Correa et al. (2024) demonstrating that nCu/PMMA reduced *Candida albicans* biofilm formation and Nurit Beyth et al. (2010) reporting a 50 % reduction in bacterial viability with QPEI-modified resins.^20^^,^^33^

However, antimicrobial effects were not always sustained over time, as shown in Jie Sun et al. (2021), where NP/PMMA lost its antimicrobial activity after 14 days of accelerated aging, raising concerns about the long-term effectiveness of silver nanoparticle incorporation.^19^ Titanium dioxide (TiO_2_) nanocomposites, assessed in two studies, demonstrated significant antimicrobial effects at lower concentrations (0.4 %) but particle aggregation at higher concentrations, potentially impacting material stability.^32^^,^^35^

From a clinical perspective, the ability of nano-modified PMMA resins to prevent microbial colonization is particularly relevant in removable prostheses, orthodontic appliances, and implant-retained dentures, where biofilm formation is a major concern. The long-term retention of antimicrobial effects, particularly in PMMA-AuNPs and QPEI-modified resins, suggests potential applications in denture wearers at high risk of denture stomatitis and patients with immuno-compromised conditions.^36^

### 4.2 Mechanical properties and structural integrity

The mechanical properties of nano-modified PMMA resins remain a subject of debate. Among the four studies that evaluated mechanical strength, findings were inconsistent.^19^^,^^32^, ^33^, ^34^ Jie Sun et al. (2021) reported a significant reduction in bending strength of NP/PMMA and NS/PMMA, suggesting that silver nanoparticles weakened the acrylic matrix.^19^ Similarly, Eugenia Eftimie Totu et al. (2017) observed that higher TiO_2_ concentrations increased viscosity and led to particle aggregation, reducing mechanical stability.^32^

In contrast, Sebastian Correa et al. (2024) found that nCu/PMMA exhibited comparable flexural strength to conventional PMMA, while Yasmin S. Zidan et al. (2025) did not explicitly report mechanical properties.^33^^,^^34^ This suggests that nanoparticle selection and concentration play a crucial role in determining the impact on material strength.

Given that mechanical durability is essential for prosthetic longevity, future studies should focus on optimizing nanoparticle integration techniques to enhance both antimicrobial activity and mechanical performance without compromising structural integrity.

### 4.3 Esthetic outcomes and patient satisfaction

Esthetic concerns remain a potential limitation of nano-modified acrylic resins. Only two studies explicitly assessed color stability and visual appearance.^15^^,^^33^ Jie Sun et al. (2021) found that NS/PMMA denture bases turned orange-yellow and NP/PMMA appeared tan indicating significant discoloration.^19^ This is a critical issue for patient satisfaction, as color changes in dental prostheses are undesirable. In contrast, Sebastian Correa et al. (2024) reported no visible esthetic differences between nCu/PMMA and conventional PMMA, suggesting that nanocopper incorporation does not impact denture appearance.^33^

While nanogold (AuNPs) and QPEI-modified resins were not explicitly assessed for esthetic properties, the absence of reported color changes in these studies suggests better color stability compared to silver-based nano-modifications. Further studies should incorporate color stability assessments over extended periods, particularly for silver and titanium-based nanoparticle-modified PMMA.

### 4.4 Material aging and long-term performance

Material aging was assessed in three studies.^19^^,^^32^^,^^33^ Jie Sun et al. (2021) observed that the antimicrobial effect of NP/PMMA disappeared after 14 days, whereas NS/PMMA retained partial inhibition effects.^19^ Eugenia Eftimie Totu et al. (2017) found that polymer matrix changes occurred with TiO_2_ addition, indicating possible structural alterations over time.^32^ Sebastian Correa et al. (2024) demonstrated that copper ion release remained within safe limits for 35 days, supporting its biocompatibility and material stability.^33^

The long-term durability of nano-modified PMMA resins is essential for clinical success, particularly for prostheses that undergo continuous mechanical and chemical stresses in the oral environment.^37^ The findings suggest that silver and titanium-based modifications may degrade more quickly, while nanocopper and QPEI-modified resins show better stability.

### 4.5 Strengths and clinical implications

A key strength of this systematic review lies in its rigorous and methodologically transparent approach to evidence synthesis. The review adhered strictly to PRISMA guidelines incorporating a comprehensive search strategy across five major databases and grey literature ensuring extensive coverage of relevant studies. The inclusion of both human and animal in vivo studies while deliberately excluding standalone in vitro studies strengthens the clinical relevance of the findings. Moreover, the application of domain-specific risk of bias tools (ROB 2.0, ROBINS-I, SYRCLE) enabled a nuanced appraisal of methodological quality across randomized trials, non-randomized studies and animal research. In addition, a structured narrative synthesis was undertaken based on the SWiM (Synthesis Without Meta-analysis) reporting guideline ensuring clarity and transparency in the absence of meta-analytic pooling.

Clinically, the findings suggest that nano-modified acrylic resins particularly those incorporating nanogold (AuNPs), nanocopper (nCu), and QPEI nanoparticles hold promising potential for use in denture bases and orthodontic appliances. These formulations were associated with sustained antimicrobial activity and favourable biocompatibility profiles. Notably, nCu-modified PMMA demonstrated effective antifungal activity without compromising mechanical strength indicating potential suitability for implant-supported prostheses. Likewise, QPEI-based modifications showed long-term microbial inhibition and material stability.

Nevertheless, silver (AgNPs) and titanium dioxide (TiO_2_)-based modifications, while antimicrobial were associated with concerns regarding mechanical degradation and esthetic alteration. This underscores the need for further material optimization and longitudinal clinical trials to validate their use in long-term applications. Thus, while promising the clinical translation of nano-modified PMMA should be approached cautiously guided by high-quality randomized evidence and standardization in evaluation protocols.

### 4.6 Limitations and future scope

This review has certain limitations. Only a limited number of in vivo studies were available, making it difficult to generalize findings to clinical settings. Additionally, heterogeneity in study designs, nanoparticle concentrations, and evaluation methods makes direct comparisons challenging. The long-term stability of antimicrobial effects also remains uncertain, as studies like Jie Sun et al. (2021) showed a decline in efficacy after 14 days of aging.^19^

Future research should focus on large-scale randomized controlled trials with extended follow-up periods to assess the long-term clinical performance of nano-modified PMMA. Additionally, standardized testing protocols are needed to evaluate the impact of different nanoparticle incorporation techniques on mechanical properties, color stability, and patient-reported outcomes. The development of composite nanomaterial formulations that balance antimicrobial efficacy with mechanical strength could further enhance the clinical applicability of nano-modified acrylic resins.

## 5 Conclusion

Nano-modified acrylic resins exhibit strong antimicrobial properties and good biocompatibility, making them promising alternatives to conventional PMMA for denture bases, implant-supported prostheses, and orthodontic appliances. However, challenges related to mechanical durability, esthetic stability, and long-term effectiveness remain. While AuNPs, QPEI, and nCu/PMMA demonstrated prolonged antimicrobial effects without significant mechanical compromise, silver and titanium-based nanoparticles require optimization due to weakened structural integrity and potential discoloration.

## Consent to participate

Not applicable.

## Patient's/guardian's consent

Since this study is a systematic review analyzing previously published in vivo studies, obtaining individual patient or guardian consent was not applicable. The data utilized in this review were extracted from publicly available studies that had already received appropriate ethical approvals from their respective institutions. No new human or animal subjects were involved in this research.

## Author contributions (CREDIT statement)

Parthasarthy Natarajan: Conceptualization, Methodology, Writing - Original Draft, Supervision.

Seenivasan Madhan Kumar: Data Curation, Formal Analysis, Writing - Review & Editing.

Shanmuganathan Natarajan: Supervision, Writing - Review & Editing.

K.S. Sridharan: Investigation, Data Curation.

S. Narayana Kalkura: Resources, Validation.

All authors have read and approved the final manuscript.

## AI declaration

No artificial intelligence (AI) tools were used in the conceptualization, writing, or editing of this manuscript.

## Ethical approval

Not applicable, as this study is a systematic review of publicly available data.

## Consent for publication

All authors provide their consent for publication.

## Availability of data and materials

Data used in this review were extracted from publicly available sources.

## Ethical clearance

This study, being a systematic review, did not involve direct experimentation on human or animal subjects and thus did not require ethical approval. All included studies were screened for prior ethical compliance, ensuring that the data used were derived from research adhering to ethical guidelines set by respective institutions and review boards.

## Funding

No funding was received for this research.

## Sources of funding

The authors confirm that this study did not receive any financial support, grants, or funding from public, commercial, or non-profit organizations. The research was conducted independently, without any financial influence that could potentially affect the interpretation of results.

## Declaration of competing interest

The authors declare that there are no conflicts of interest related to this study. No financial, personal, or professional relationships influenced the conduct, analysis, or reporting of this systematic review. The content presented reflects an unbiased synthesis of existing in vivo evidence on nano-particle coated or impregnated acrylic resins in dental applications.
