# Supplementary material for: Nano-particle coated or impregnated acrylic resins in dental applications: A systematic review of in Vivo Evidence on mechanical properties, biocompatibility and clinical performance
Source: J Oral Biol Craniofac Res. 2025 Aug 7;15(6):1190–9. doi: 10.1016/j.jobcr.2025.07.018 (PMC12355065; doi:10.1016/j.jobcr.2025.07.018)
Supplement: Multimedia component 1 [file mmc1.docx]

# **Supplementary table 1: Search Strategy Table**

| Database | Modified Search Terms | Filters Applied | Search Date | Number of Articles Retrieved |
| --- | --- | --- | --- | --- |
| PubMed/MEDLINE | "Acrylic resin" OR "Polymethyl methacrylate" OR "Denture base resin" AND ("Nano-particle" OR "Nano-coating" OR "Nano-impregnation" OR "Nano-filler") AND ("Mechanical properties" OR "Flexural strength" OR "Fracture resistance" OR "Wear resistance" OR "Biocompatibility" OR "Cytotoxicity" OR "In vivo toxicity" OR "Host response" OR "Antimicrobial activity" OR "Biofilm inhibition" OR "Bacterial adhesion" OR "Esthetics" OR "Color stability" OR "Surface roughness" OR "Gloss retention" OR "Material aging" OR "Degradation" OR "Durability" OR "Allergic reactions" OR "Inflammation" OR "Immune response") | Human and animal studies, English language, Full-text available, Peer-reviewed journals | Until 18-01-2025 | 5 |
| Scopus | TS=("Acrylic resin" OR "Polymethyl methacrylate" OR "Denture base resin") AND ("Nano-particle" OR "Nano-coating" OR "Nano-impregnation" OR "Nano-filler") AND ("Mechanical properties" OR "Flexural strength" OR "Fracture resistance" OR "Wear resistance" OR "Biocompatibility" OR "Cytotoxicity" OR "In vivo toxicity" OR "Host response" OR "Antimicrobial activity" OR "Biofilm inhibition" OR "Bacterial adhesion" OR "Esthetics" OR "Color stability" OR "Surface roughness" OR "Gloss retention" OR "Material aging" OR "Degradation" OR "Durability" OR "Allergic reactions" OR "Inflammation" OR "Immune response") | Human and animal studies, English language, Full-text available, Clinical trials | Until 18-01-2025 | 82 |
| Web of Science | ALL fields =("Acrylic resin" OR "Polymethyl methacrylate" OR "Denture base resin") AND ("Nano-particle" OR "Nano-coating" OR "Nano-impregnation" OR "Nano-filler") AND ("Mechanical properties" OR "Flexural strength" OR "Fracture resistance" OR "Wear resistance" OR "Biocompatibility" OR "Cytotoxicity" OR "In vivo toxicity" OR "Host response" OR "Antimicrobial activity" OR "Biofilm inhibition" OR "Bacterial adhesion" OR "Esthetics" OR "Color stability" OR "Surface roughness" OR "Gloss retention" OR "Material aging" OR "Degradation" OR "Durability" OR "Allergic reactions" OR "Inflammation" OR "Immune response") | Human and animal studies, English language, Full-text available, Controlled trials | Until 18-01-2025 | 9 |
| Cochrane Library | ("Acrylic resin" OR "Polymethyl methacrylate" OR "Denture base resin") AND ("Nano-particle" OR "Nano-coating" OR "Nano-impregnation" OR "Nano-filler") AND ("Mechanical properties" OR "Flexural strength" OR "Fracture resistance" OR "Wear resistance" OR "Biocompatibility" OR "Cytotoxicity" OR "In vivo toxicity" OR "Host response" OR "Antimicrobial activity" OR "Biofilm inhibition" OR "Bacterial adhesion" OR "Esthetics" OR "Color stability" OR "Surface roughness" OR "Gloss retention" OR "Material aging" OR "Degradation" OR "Durability" OR "Allergic reactions" OR "Inflammation" OR "Immune response") | Human and animal studies, English language, Full-text available, Experimental studies | Until 18-01-2025 | 1 |
| Embase | ("Acrylic resin" OR "Polymethyl methacrylate" OR "Denture base resin") AND ("Nano-particle" OR "Nano-coating" OR "Nano-impregnation" OR "Nano-filler") AND ("Mechanical properties" OR "Flexural strength" OR "Fracture resistance" OR "Wear resistance" OR "Biocompatibility" OR "Cytotoxicity" OR "In vivo toxicity" OR "Host response" OR "Antimicrobial activity" OR "Biofilm inhibition" OR "Bacterial adhesion" OR "Esthetics" OR "Color stability" OR "Surface roughness" OR "Gloss retention" OR "Material aging" OR "Degradation" OR "Durability" OR "Allergic reactions" OR "Inflammation" OR "Immune response") | Human and animal studies, English language, Full-text available, Preclinical research | Until 18-01-2025 | 1057 |

# **Supplementary table 2: Articles excluded with reasons**

| S. No. | Citation | Reason for Exclusion |
| --- | --- | --- |
| 1 | Menon RK et al. CADCAM versus conventional denture bases: network meta-analysis of in vitro studies. Int Dent J. 2025. | Excluded - Systematic review of in vitro studies |
| 2 | Monteiro DR et al. Antimicrobial effect of medical devices containing silver. Int J Antimicrob Agents. 2009. | Excluded - In vitro study |
| 3 | Sharma A et al. Role of nanoparticles in denture base resins: A review. J Oral Biol Craniofac Res. 2022. | Excluded - Review article |
| 4 | Malik S, Waheed Y. Emerging Applications of Nanotechnology in Dentistry. Dent J (Basel). 2023. | Excluded - Review article |
| 5 | Leão RS et al. Influence of zirconia on PMMA: A systematic review. Mater Sci Eng C. 2020. | Excluded - Systematic review |
| 6 | Alzayyat ST et al. Effects of SiO2 on denture base resin. Eur J Dent. 2022. | Excluded - In vitro study |
| 7 | Gligorijević N et al. Antimicrobial Properties of Silver-Modified Resins. Nanomaterials. 2022. | Excluded - In vitro study |
| 8 | Thambirajoo M et al. Nanoparticles with antibacterial properties. Antibiotics. 2021. | Excluded - Review article |
| 9 | Weisman D et al. Antibiotic elution from PMMA. Vet Surg. 2000. | Excluded - In vitro study |
| 10 | Moffa EB et al. Antimicrobial activity of PMMA enriched with nano-clay. Braz Oral Res. 2024. | Excluded - In vitro study |
| 11 | Lassila L et al. Properties of 3D-printed dental resin. Polymers. 2024. | Excluded - In vitro study |
| 12 | Goiato MC et al. Accelerated aging of resins. Braz Oral Res. 2010. | Excluded - In vitro study |
| 13 | Papathanasiou I et al. Color stability of dental prosthetics. J Prosthodont. 2021. | Excluded - In vitro study |
| 14 | Gad MM et al. Optical properties of PMMA with nanoparticles. Int J Nanomedicine. 2017. | Excluded - In vitro study |
| 15 | Zakia M et al. Silver nanoparticle-based hydrogel. Green Chem Lett Rev. 2020. | Excluded - In vitro study |
| 16 | Lourinho C et al. Mechanical Properties of PMMA: Meta-analysis. Biomedicines. 2022. | Excluded - Systematic review of in vitro studies |
| 17 | Pourhajibagher M et al. Nanoparticles in acrylic resins. J Oral Biol Craniofac Res. 2022. | Excluded - Meta-analysis of in vitro studies |
| 18 | Kaurani P et al. TiO2 in PMMA: Systematic Review. F1000Res. 2022. | Excluded - Systematic review of in vitro studies |
| 19 | Trial ID: CTRI/2022/08/044721. Evaluation of microbial accumulation in denture wearers. WHO ICTRP. 2022. | Excluded - Ongoing trial, no published results yet |
| 20 | Nam KY et al. Effects of incorporating silver nanoparticles into denture acrylic resin. J Prosthet Dent. 2012. | Excluded - In vitro study |
| 21 | Acosta-Torres LS et al. Biocompatibility and antifungal activity of PMMA-silver nanoparticle composites. Mater Sci Eng C. 2012. | Excluded - In vitro study |
| 22 | Monteiro DR et al. Silver-containing resins: dispersion, release and antifungal activity. Int J Antimicrob Agents. 2012. | Excluded - In vitro study |
| 23 | Hamedi-Rad F et al. PMMA reinforced with nanosilver: an in vitro evaluation. J Dent Res Dent Clin Dent Prospects. 2014. | Excluded - In vitro study |
| 24 | Ghaffari T et al. Impact of silver nanoparticles on PMMA resin. J Prosthodont Res. 2014. | Excluded - In vitro study |
| 25 | Kim et al. Toxicity of zinc oxide nanoparticles. J Appl Toxicol. 2014. | Excluded - In vitro/animal toxicology study |
| 26 | Padmavathy N et al. Antibacterial properties of ZnO nanoparticles. Sci Technol Adv Mater. 2008. | Excluded - In vitro study |
| 27 | Nair S et al. Influence of ZnO nanoparticle size and shape on toxicity. J Biomed Nanotechnol. 2009. | Excluded - In vitro study |
| 28 | Abd ST et al. Anti-Candida efficacy of ZnO nanoparticles. Med J Islamic World Acad Sci. 2015. | Excluded - In vitro study |
| 29 | Cierech M et al. Incorporation of ZnO nanoparticles into PMMA. Dent Mater. 2016. | Excluded - In vitro study |
| 30 | Cierech M et al. Antifungal PMMA-ZnONPs composites. Dent Mater J. 2016. | Excluded - In vitro study |
| 31 | Japil D et al. Thermal degradation of PMMA with ZnO. Polym Degrad Stab. 2016. | Excluded - In vitro study |

**Supplementary figure 1: Summary ROB plot for ROB 2.0 tool**

**
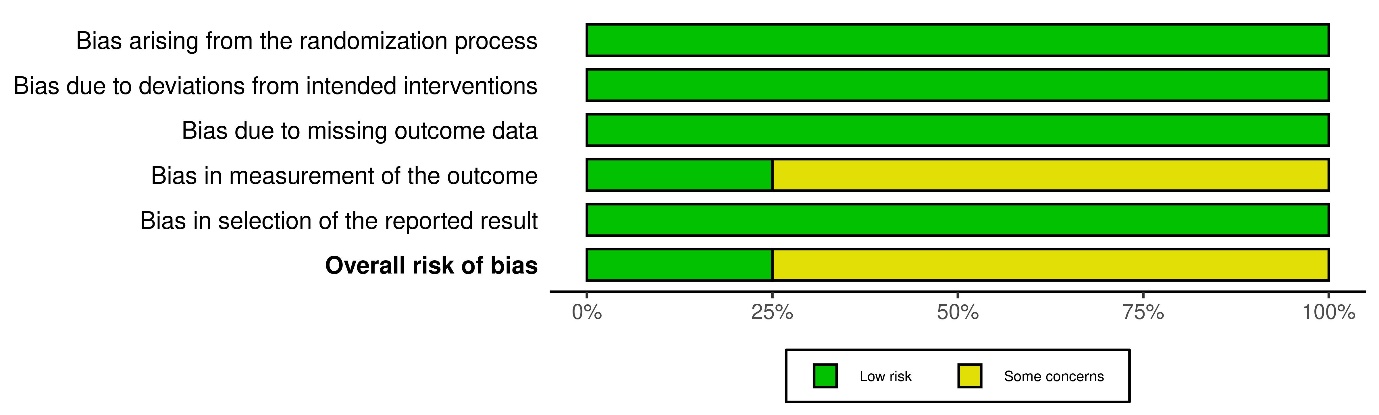
**

**Supplementary figure 2: Summary ROB plot for ROBINS-I tool**

**
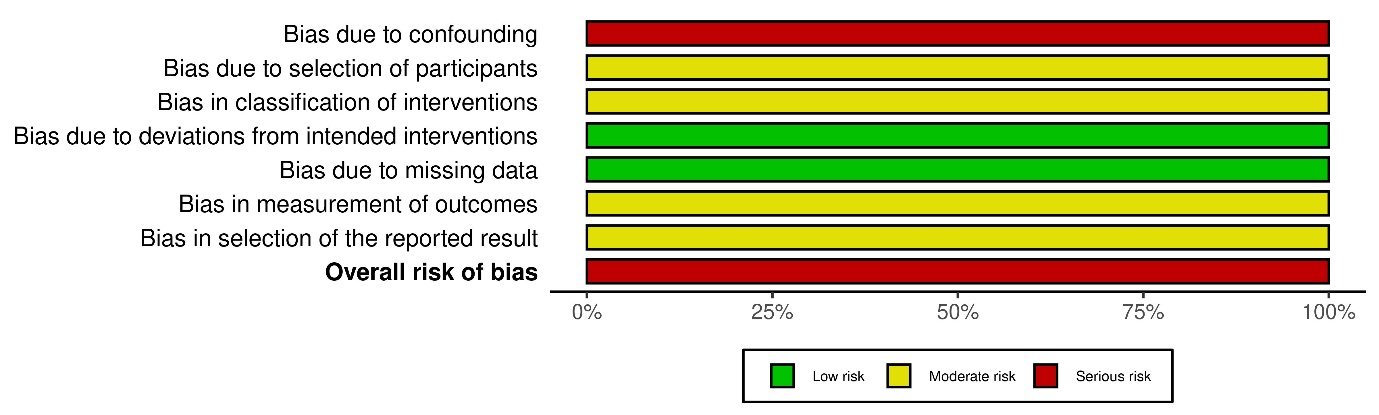
**

**Supplementary figure 3: Summary ROB plot for SYRCLE**

**
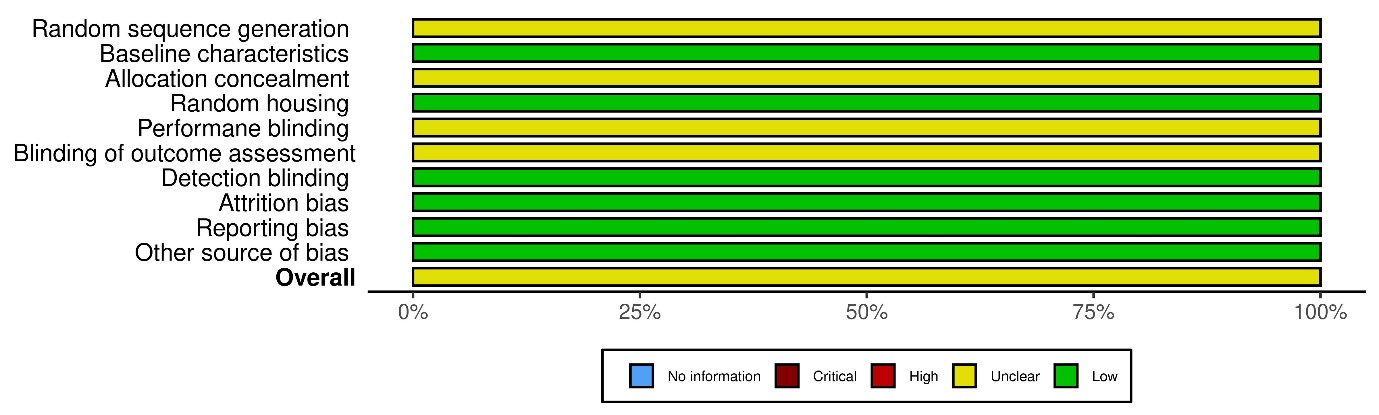
**

**Supplementary figure 4: Summary ROB plot for QUIN tool**

**
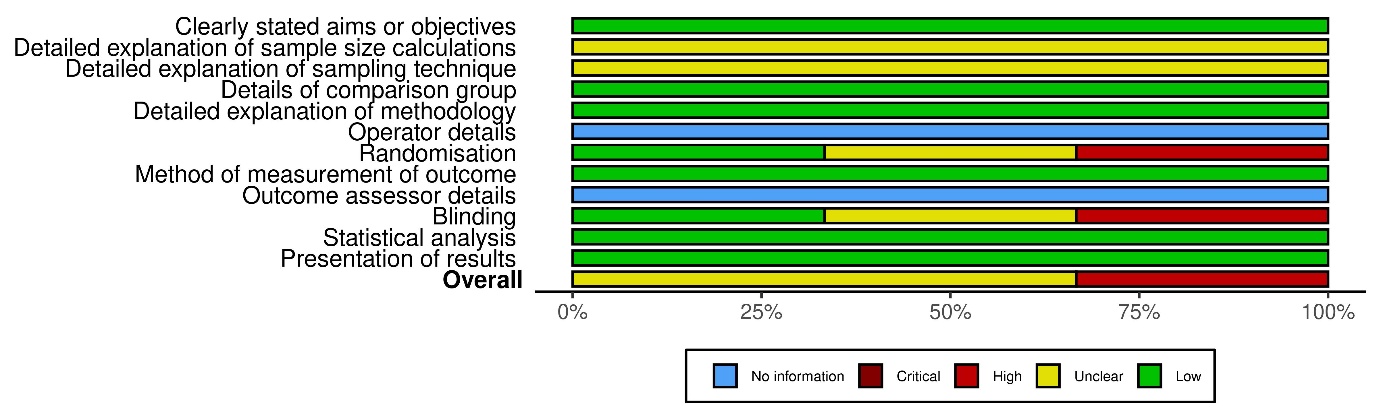
**
